# Compound Kushen injection plus platinum-based chemotherapy for stage IIIB/IV non-small cell lung cancer: A protocol for meta-analysis of randomized clinical trials following the PRISMA guidelines

**DOI:** 10.1097/MD.0000000000018552

**Published:** 2019-12-27

**Authors:** Hongwei Chen, Xiaojun Yao, Ting Li, Christopher Wai-Kei Lam, Huixia Zhang, Jue Wang, Wei Zhang, Elaine Lai-Han Leung, Qibiao Wu

**Affiliations:** a State Key Laboratory of Quality Research in Chinese Medicine; b Faculty of Medicine; c Faculty of Chinese Medicine, Macau University of Science and Technology, Macau, China.

**Keywords:** compound Kushen injection, meta-analysis, non-small-cell lung carcinoma (NSCLC), platinum-based chemotherapy, protocol, systematic review

## Abstract

**Background:**

Compound Kushen injection (CKI) is a commonly used anti-tumor Chinese patent medicine, which is extracted from Kushen (*Radix Sophorae* Flavescentis) and Baituling (*Rhizoma Smilacis* Glabrae) and has been widely prescribed as an add-on therapy to platinum-based chemotherapy (PBC) for advanced non-small cell lung cancer (NSCLC). However, the efficacy and safety of this combination therapy remain controversial.

**Methods and analysis:**

A systematic review and meta-analysis will be performed following the PRISMA (Preferred Reported Items for Systematic Review and Meta-analysis) guidelines. All randomized controlled trials (RCTs) comparing CKI in combination with PBC versus PBC alone will be retrieved and assessed for inclusion. Analyses will be performed using Review Manager 5.3, Comprehensive Meta-Analysis 3.0 and Trial Sequential Analysis software. The disease control rate (DCR) will be defined as the primary outcome, and the objective response rate (ORR), quality of life (QOL), survival rate, and toxicities will be the secondary outcomes.

**Results:**

This study will systematically evaluate the efficacy and safety of Compound Kushen injection combined with platinum-based chemotherapy in the treatment of stage III/IV NSCLC. The results of this study will be published in a peer-reviewed journal.

**Conclusions:**

This systematic review and meta-analysis of eligible randomized controlled trials will evaluate the effects of Compound Kushen injection as adjunctive therapy to platinum-based chemotherapy in patients with stage III/IV non-small cell lung cancer, thus providing evidence to the clinical use of this combination therapy for the specific subsets of patients.

**PROSPERO registration number:**

CRD42019134892

## 1 Introduction

Worldwide, lung cancer remains the most common cancer and the leading cause of cancer-related mortality.[^1^,^2^,^3^] In 2018, there were about 2.1 million new lung cancer cases and 1.8 million deaths.[^1^,^2^,^3^] The incidence and mortality rates of lung cancer have rapidly increased in recent years.[^1^,^4^,^5^] Non-small cell lung cancer (NSCLC) accounts for approximately 85% of all lung cancer and the majority of newly diagnosed NSCLC patients are already at stage III/IV.^[2]^

Platinum-based chemotherapy (PBC) is a commonly recommended treatment choice for the advanced NSCLC patients, especially for those not treated with targeted therapy and immunotherapy due to lack of an actionable biomarker or unavailability of precision therapies.[^6^,^7^,^8^,^9^,^10^] The prognosis of stage IIIB/IV NSCLC is extremely poor with a median survival of 7.9 months,^[11]^ and compared with precision therapies, the treatment with PBC alone is usually associated with worse survival, poor quality of life (QOL), and increased risk of toxic effects.[^7^,^9^,^12^] Therefore, it is necessary to find better therapy to improve the outcomes and reduce adverse events in the advanced NSCLC patients treated with PBC.

In China and some Asian countries, traditional Chinese medicines have been increasingly prescribed for late-stage lung cancer in combination with PBC for synergistic interactions.[^13^,^14^] Compound Kushen injection (CKI) is one of the anti-tumor Chinese patent medicines approved by the State Food and Drug Administration of China (Drug Approval Number: Z14021231) for the management of various cancers.^[15]^ CKI is a mixture of natural compounds extracted from two medicinal herbs, Kushen (*Radix Sophorae* Flavescentis) and Baituling (*Rhizoma Smilacis* Glabrae). Matrine, oxymatrine, sophoridine, and N-methylcytisine are the active ingredients of CKI.[^16^,^17^] Emerging studies have shown that CKI and its active ingredients have significant anti-tumor activities,[^17^,^18^,^19^] such as inhibiting cancer cell proliferation, invasion and metastasis,[^16^,^20^,^21^] inducing tumor cell apoptosis,[^22^,^23^] reducing angiogenesis,^[21]^ inducing cell cycle arrest,[^20^,^21^,^23^] inhibiting glycometabolism and amino acid metabolism,^[16]^ and overcoming multidrug resistance to chemotherapy.[^24^,^25^] Besides, CKI can effectively enhance immunologic function^[26]^ and alleviate chemoradiotherapy-induced toxicity.[^21^,^24^,^25^]

A growing number of randomized controlled trials (RCTs) have investigated the effectiveness of CKI as an add-on therapy to PBC for advanced NSCLC patients, however, the results were inconsistent and the conclusion remains unclear. Some RCTs suggested that this combination therapy improved clinical effectiveness and reduce adverse events,[^18^,^19^] but some found no significant changes in the relevant outcomes.[^27^,^28^,^29^,^30^,^31^,^32^] The effects of CKI combined with PBC for patients with stage III/IV NSCLC have never been systematically evaluated. Therefore, it is necessary to assess the efficacy and safety of CKI combined with PBC for patients with stage III/IV NSCLC, aiming to provide optimal therapy for the specific subsets of patients.

## 2 Method

### 2.1 Study registration

This study has been registered as PROSPERO CRD42019134892 (https://www.crd.york.ac.uk/prospero/display_record.php?RecordID=134892). This systematic review and meta-analysis will be performed following the PRISMA (Preferred Reported Items for Systematic Review and Meta-analysis) guidelines.^[33]^ Because all the research materials are published studies, ethical approval is not necessary.

### 2.2 Criteria for considering studies for this review

#### 2.2.1 Types of studies

All RCTs comparing CKI plus PBC versus PBC alone will be selected and assessed for inclusion in this study, regardless of language, publication status, and publication year.

#### 2.2.2 Types of participants

The participants included in this study should meet the following criteria: Diagnosis of stage III-IV NSCLC using the histopathological/cytological diagnostic criteria and TNM staging system[^34^,^35^,^36^] at least one bi-dimensionally measurable lesion; Karnofsky performance status (KPS) score^[37]^ of at least 60; the range of Performance Status score from 0 to 2; life expectancy at least 3 months. The gender, age, and ethnicity of the patients are not limited.

#### 2.2.3 Types of interventions

The intervention in the experimental groups was CKI plus PBC; the intervention in the control groups was PBC only. In each study, the PBC regimen used in both experimental and control groups was the same. All PBC regimens were eligible.

#### 2.2.4 Types of outcome measures

The primary clinical endpoint will be the disease control rate (DCR). Objective response rate (ORR), QOL, and toxic effects will be defined as the secondary outcomes. According to the WHO criteria for reporting results of cancer treatment,[^38^,^39^] ORR and DCR will be used to assess the short-term effectiveness. Improvement of QOL will be considered when KPS score increased by 10 points or more after treatment. Anti-tumor drug toxicity will be evaluated and classified as grades 0 to 4, according to Recommendations for Grading of Acute and Subacute Toxicity.^[38]^ In this research, grades 3 and 4 toxicities will be defined as severe toxicities.

### 2.3 Search methods for the identification of studies

#### 2.3.1 Search strategy

A comprehensive literature search was conducted by 2 independent researchers (HWC and HXZhang). Common databases (PubMed, Web of Science, ClinicalTrials.gov, Cochrane Library, EMBASE, Wanfang Databases, China National Knowledge Infrastructure, the Chinese Scientific Journal Database, the Chinese Science Citation Database, and the Chinese Biomedical Literature Database) were retrieved from inception to April 20, 2019. The search details were conducted as follows (English database): {(“Carcinoma, Non-Small-Cell Lung”[MeSH] OR “Carcinoma, Non-Small-Cell Lung” [Title/Abstract] OR “Carcinomas, Non-Small-Cell Lung” [Title/Abstract] OR “Lung Carcinoma, Non-Small-Cell” [Title/Abstract] OR “Lung Carcinomas, Non-Small-Cell” [Title/Abstract] OR “Non-Small-Cell Lung Carcinomas” [Title/Abstract] OR “Non-small Cell Lung Cancer” [Title/Abstract] OR “Non-Small-Cell Lung Carcinoma” [Title/Abstract] OR “Non-Small Cell Lung Carcinoma” [Title/Abstract] OR “Carcinoma, Non-Small Cell Lung” [Title/Abstract] OR “Non-Small Cell Lung Cancer” [Title/Abstract]) AND (“Compound Kushen injection” [Title/Abstract] OR “Fufang Kushen injection” [Title/Abstract] OR “Yanshu injection”)}. Chinese databases (CNKI, etc.) searches: {“feixiaoxibaofeiai” (“carcinoma, non-small-cell lung”) AND (“Fufangkushenzhusheye” (“Compound Kushen injection” OR “Fufang Kushen injection” OR “Yanshu injection”) AND Hualiao (“chemotherapy”)}. Besides, the relevant systematic reviews and meta-analyses were searched and evaluated to find potential RCTs from their references.

#### 2.3.2 Study selection

Two independent reviewers (HWC and TL) screened the titles and abstracts of all the articles for eligible studies. The full texts were retrieved for further assessment according to the inclusion and exclusion criteria. All disagreements were resolved by consensus.

#### 2.3.3 Data collection and management

Three reviewers (HWC, XJY, and HXZ) will independently rate the included RCTs and extract the data. If a trial reported ambiguous or incomplete data, reviewers will contact the corresponding author via email and/or phone for further information. The intention-to-treat (ITT) analysis will be used to analyze the results whenever available. Any disagreement will be resolved by discussing with a third reviewer (QBW).

### 2.4 Assessment of risk of bias in included studies

Two independent reviewers (HWC and TL) will appraise the risk of bias in the included trials using the Cochrane Risk of Bias Tool for Randomized Controlled Trials.^[38]^ The following criteria will be used to evaluate bias in each trial: random sequence generation; concealment of allocation; blinding of participants and personnel; blinding of outcome assessment; incomplete data; selective reporting; and other bias. The risk of bias will be classified as “unclear”, “low” or “high”.

#### 2.4.1 Measurement of treatment effect

Dichotomous data will be shown as the risk ratio (RR), risk difference (RD) or odds ratio (OR), and continuous data will be presented as the weighted mean difference (WMD) or standardized mean difference (SMD) with 95% confidence intervals (CI).[^40^,^41^,^42^]

#### 2.4.2 Assessment of heterogeneity

Heterogeneity will be assessed using the *I* ^2^ statistic and Chi^2^ test. Substantial heterogeneity will be considered when *I* ^2^ > 50% or *P* < .1.[^40^,^41^,^42^,^43^]

#### 2.4.3 Data synthesis

All analyses will be performed using the Review Manager (RM) 5.3 (Copenhagen: The Nordic Cochrane Centre, The Cochrane Collaboration, 2014), the Comprehensive Meta-Analysis (CMA) 3.0 (Biostat, Englewood, NJ, 2016) and the Trial Sequential Analysis (TSA) software (Copenhagen Trial Unit, Centre for Clinical Intervention Research, Copenhagen, Denmark; 2011). If the hypothesis of homogeneity is not rejected, a fixed-effects model will be used to estimate the summary RR (OR or RD), WMD (or SMD) and 95% CI; otherwise, a random-effects model will be used.[^40^,^41^,^42^,^43^,^44^] If quantitative synthesis is inappropriate, we will provide a systematic narrative synthesis with the information presented to summarize and explain the characteristics and findings of the included studies.^[45]^ The strength of the evidence will be evaluated using the Grading of Recommendations Assessment, Development and Evaluation working group methodology (GRADE).[^46^,^47^]

#### 2.4.4 Risk of Bias across trials

When the number of the included trials is >10, funnel plots and Egger test will be used to examine the potential bias in the RCTs included in the meta-analysis.[^48^,^49^]

#### 2.4.5 Additional analyses

Sensitivity analysis, subgroup analysis and the Trial Sequential Analysis (TSA) will be used to determine the robustness of results and calculate the required information size (RIS) in the meta-analysis.^[50]^ A meta-regression analysis will be implemented to determine the potential heterogeneity and the impact of moderator variables on the study effect size.

## 3 Discussion

So far, there is no published systematic review and meta-analysis evaluating the efficacy and safety of Compound Kushen injection plus PBC for stage III/IV NSCLC, this study will systematically evaluate the effects of Compound Kushen injection as an add-on therapy to PBC in the treatment of stage III/IV NSCLC, thus providing some evidence to the clinical use of this combination therapy for the specific subsets of patients.

## Author contributions

**Conceptualization:** Qibiao Wu, Xiaojun Yao, Ting Li, Elaine Lai-Han Leung.

**Data curation:** Qibiao Wu, Hongwei Chen, Xiaojun Yao.

**Formal analysis:** Qibiao Wu, Hongwei Chen, Ting Li.

**Funding acquisition:** Qibiao Wu, Xiaojun Yao, Elaine Lai-Han Leung.

**Investigation:** Qibiao Wu, Hongwei Chen, Xiaojun Yao, Ting Li, Huixia Zhang, Jue Wang.

**Methodology:** Qibiao Wu, Hongwei Chen, Xiaojun Yao.

**Project administration:** Qibiao Wu, Xiaojun Yao.

**Resources:** Qibiao Wu, Wei zhang.

**Software:** Qibiao Wu, Hongwei Chen, Xiaojun Yao, Huixia Zhang.

**Supervision:** Qibiao Wu, Xiaojun Yao, Elaine Lai-Han Leung.

**Validation:** Qibiao Wu, Hongwei Chen, Xiaojun Yao, Ting Li, Christopher Wai-Kei Lam, Huixia Zhang, Jue Wang, Wei zhang, Elaine Lai-Han Leung.

**Visualization:** Qibiao Wu.

**Writing – original draft:** Qibiao Wu, Hongwei Chen, Ting Li.

**Writing – review & editing:** Qibiao Wu, Xiaojun Yao, Christopher Wai-Kei Lam, Elaine Lai-Han Leung.

Elaine Lai-Han Leung orcid: 0000-0002-3705-8084.

Qibiao Wu orcid: 0000-0002-1670-1050.
